# Nutritional Management in Patients With Hematological Malignancies: An Underestimated Supportive Care Issue

**DOI:** 10.7759/cureus.104677

**Published:** 2026-03-04

**Authors:** Yasmine El Ouafa, Monsif Fadi, Houda Benrahma, Nouama Bouanani

**Affiliations:** 1 Center for Doctoral Studies (CeDoc), Mohammed VI University of Health Sciences (UM6SS), Casablanca, MAR; 2 Interdisciplinary Laboratory of Biotechnology and Health, Mohammed VI University of Health Sciences (UM6SS), Casablanca, MAR; 3 Center for Doctoral Studies (CeDoc), Oncopathology, Cancer Biology and Environment Laboratory, Mohammed VI Faculty of Medicine, Mohammed VI University of Health Sciences (UM6SS), Casablanca, MAR; 4 Medicine, Mohammed VI University of Health Sciences (UM6SS), Casablanca, MAR; 5 Center for Doctoral Studies (CeDoc), Interdisciplinary Laboratory of Biotechnology and Health, Mohammed VI University of Health Sciences (UM6SS), Casablanca, MAR; 6 Hematology, Faculty of Medicine, Mohammed VI University of Health Sciences (UM6SS), Casablanca, MAR

**Keywords:** hematological malignancies, malnutrition, morocco, nutritional assessment, supportive care

## Abstract

Advances in the management of hematological malignancies have significantly improved survival outcomes; however, supportive care remains a critical determinant of treatment tolerance and overall prognosis. Malnutrition is highly prevalent among patients with hematological cancers and is associated with increased treatment-related toxicity, infectious complications, prolonged hospitalization, and reduced quality of life. In Morocco, nutritional management in hematology is frequently under-recognized and inconsistently implemented. Limited availability of specialized clinical nutrition services, insufficient systematic nutritional screening, and the absence of national guidelines contribute to heterogeneous practices across institutions. Moreover, treatment-related gastrointestinal toxicities, prolonged hospital stays, and socioeconomic constraints further exacerbate nutritional deterioration in this vulnerable population. This editorial highlights the current burden of malnutrition among Moroccan patients with hematological malignancies, reviews available national data, and discusses organizational and structural barriers to optimal nutritional care. Addressing these gaps through early nutritional assessment, multidisciplinary collaboration, and the integration of standardized nutritional protocols is essential to improve treatment tolerance, clinical outcomes, and equity of supportive care in hematology in Morocco.

## Editorial

Recent therapeutic advances in hematological malignancies have markedly improved survival; however, gaps in supportive care, particularly nutritional management, continue to compromise treatment tolerance and overall prognosis. Malnutrition affects up to 40-50% of patients with cancer and is frequently under-recognized in hematological practice, despite its association with increased treatment-related toxicity, infectious complications, prolonged hospitalization, and impaired quality of life [1]. In Morocco, nutritional management in hematology is frequently under-recognized and inconsistently implemented. Limited availability of specialized clinical nutrition services, insufficient systematic nutritional screening, and the absence of national guidelines contribute to heterogeneous practices across institutions. Moreover, treatment-related gastrointestinal toxicities, prolonged hospital stays, and socioeconomic constraints further exacerbate nutritional deterioration in this vulnerable population. This editorial highlights the current burden of malnutrition among Moroccan patients with hematological malignancies, reviews available national data, and discusses organizational and structural barriers to optimal nutritional care. Addressing these gaps, particularly in systematic nutritional screening, access to clinical nutrition specialists, and early intervention in hematology units through collaboration among hematologists, dietitians, nurses, and pharmacists, and by implementing standardized nutritional protocols in tertiary hospitals, is essential to improve treatment tolerance, clinical outcomes, and equity of supportive care in Morocco.

Advances in the treatment of hematological malignancies have led to substantial improvements in survival over recent decades, notably through the introduction of targeted therapies, immunotherapies, and modern hematopoietic stem cell transplantation strategies. However, these therapeutic gains are frequently undermined by insufficient attention to supportive care, particularly nutritional management. Malnutrition remains highly prevalent among patients with hematological cancers and continues to be under-recognized in routine clinical practice, despite its well-established association with treatment intolerance, infectious complications, prolonged hospitalization, and impaired quality of life [2].

In Morocco, cancer represents a major public health concern, with more than 50 000 new cases diagnosed annually according to the most recent estimates from the Moroccan Association of Cancer Registries (AMRC) based on aggregated data from regional cancer registries and national reporting systems. Hematological malignancies, including leukemias, lymphomas, and multiple myeloma, account for a meaningful proportion of this burden, although precise national epidemiological data remain limited due to incomplete cancer registries. Available regional data indicate that lymphomas represent approximately 5-6 % of all cancers, while leukemias account for nearly 2-3% [3]. Malnutrition is common among Moroccan patients with cancer, with reported prevalence rates ranging from 40% to nearly 50% in adult oncology populations, and up to 40% in pediatric patients with malignancies at diagnosis [4]. These figures, although derived largely from oncology units, may reflect a similar or even greater vulnerability among patients with hematological malignancies, given the higher metabolic demands, treatment intensity, and immunosuppression in this population. Comparable prevalence rates have been reported in regional studies from other North African countries and in global indices, where malnutrition among hematology patients ranges between 40 and 60% at diagnosis, highlighting a consistent risk pattern across similar settings [5].

In daily hematology practice, nutritional risk is often underestimated and frequently unrecognized. This occurs because standard clinical measures, such as body mass index, may appear normal or elevated, masking underlying sarcopenia, micronutrient deficiencies, or significant metabolic stress. Moreover, subtle declines in nutritional status may develop insidiously and remain undiagnosed without systematic screening. As a result, many individuals begin intensive chemotherapy, targeted therapies, or conditioning regimens for hematopoietic stem cell transplantation in a nutritionally fragile state. This hidden vulnerability contributes to dose reductions, treatment delays, increased susceptibility to infections, and prolonged exposure to supportive therapies such as antibiotics. These associations are supported by evidence demonstrating that malnutrition and nutritional risk are linked with adverse clinical outcomes in patients with hematological malignancies and other cancer populations [5]. Beyond its clinical consequences, inadequate nutritional care increases healthcare costs and places additional strain on already limited hospital resources.

Several organizational and structural barriers further complicate nutritional management in Moroccan hematology units. These barriers are derived from institutional experience and expert clinical consensus and are consistent with challenges reported in the broader Moroccan oncology context, although formal nationwide data in hematology populations remain limited. Nutritional screening is not systematically implemented, access to clinical nutrition specialists is limited, and dietitians are often not integrated into hematology teams. This observation is consistent with evidence from broader oncology care settings demonstrating wide variability and underutilization of nutritional screening in clinical practice. For example, international surveys show that nutritional screening is performed in only a minority of cancer centers, and barriers such as lack of standardized protocols, limited access to dietitians, and resource constraints hinder systematic implementation, particularly in low- and middle-income settings [6]. Nutritional interventions are often delayed rather than incorporated early as part of the therapeutic strategy. In addition, the absence of national guidelines dedicated to nutritional care in hematological malignancies leads to heterogeneous practices between institutions. Socioeconomic constraints, limited availability of nutritional supplements, and insufficient training of healthcare professionals in clinical nutrition further contribute to suboptimal care. In this context, patients and their families may turn to non-medical dietary restrictions or traditional beliefs, which can unintentionally worsen nutritional intake and add to psychological distress.

Addressing malnutrition in hematological malignancies requires a shift in perspective, recognizing nutritional care as an integral component of cancer treatment rather than a secondary consideration [7]. Systematic nutritional screening at diagnosis and during follow-up, using validated tools adapted to hematology such as the Malnutrition Universal Screening Tool (MUST), Nutritional Risk Screening 2002 (NRS-2002), and the Patient-Generated Subjective Global Assessment (PG-SGA), allows early identification of patients at risk and timely intervention before irreversible deterioration occurs [8-10]. Strengthening multidisciplinary collaboration between hematologists, dietitians, nurses, and pharmacists is essential to ensure individualized and continuous nutritional support [7]. Even in resource-limited settings, simplified nutritional pathways and structured assessment protocols may substantially improve clinical outcomes.

All patients with cancer should benefit from regular nutritional screening and a comprehensive assessment encompassing dietary intake, body composition, symptoms interfering with nutrition, muscle mass, physical performance, and markers of systemic inflammation throughout the disease course. In this context, the development of adapted guidelines, based on established international recommendations for the nutritional management of hematological malignancies and tailored to the Moroccan healthcare context, together with targeted training programs for healthcare professionals, would represent a crucial step toward standardizing practices and reducing inequalities in care. Early nutritional intervention, initiated at or near the time of cancer diagnosis or the start of therapy, can improve nutritional status, attenuate weight loss, enhance tolerance to anticancer treatments, and reduce treatment-related complications. Strengthening nutritional management should not be viewed solely as an organizational or logistical issue; rather, it constitutes a fundamental and evidence-based component of standard oncologic care, directly influencing treatment tolerance, functional capacity, and overall quality of life in patients with hematological malignancies.
